# The Effect of Modality and Narration Style on Recall of Online Health Information: Results From a Web-Based Experiment

**DOI:** 10.2196/jmir.4164

**Published:** 2015-04-24

**Authors:** Nadine Bol, Julia CM van Weert, Hanneke CJM de Haes, Eugene F Loos, Ellen MA Smets

**Affiliations:** ^1^Amsterdam School of Communication Research / ASCoRDepartment of Communication ScienceUniversity of AmsterdamAmsterdamNetherlands; ^2^Department of Medical PsychologyAcademic Medical CenterUniversity of AmsterdamAmsterdamNetherlands

**Keywords:** instructional films and videos, narration, personal narratives, age groups, consumer health information, memory, mental recall, patient education

## Abstract

**Background:**

Older adults are increasingly using the Internet for health information; however, they are often not able to correctly recall Web-based information (eHealth information). Recall of information is crucial for optimal health outcomes, such as adequate disease management and adherence to medical regimes. Combining effective message strategies may help to improve recall of eHealth information among older adults. Presenting information in an audiovisual format using conversational narration style is expected to optimize recall of information compared to other combinations of modality and narration style.

**Objective:**

The aim of this paper is to investigate the effect of modality and narration style on recall of health information, and whether there are differences between younger and older adults.

**Methods:**

We conducted a Web-based experiment using a 2 (modality: written vs audiovisual information) by 2 (narration style: formal vs conversational style) between-subjects design (N=440). Age was assessed in the questionnaire and included as a factor: younger (<65 years) versus older (≥65 years) age. Participants were randomly assigned to one of four experimental webpages where information about lung cancer treatment was presented. A Web-based questionnaire assessed recall of eHealth information.

**Results:**

Audiovisual modality (vs written modality) was found to increase recall of information in both younger and older adults (*P*=.04). Although conversational narration style (vs formal narration style) did not increase recall of information (*P*=.17), a synergistic effect between modality and narration style was revealed: combining audiovisual information with conversational style outperformed combining written information with formal style (*P*=.01), as well as written information with conversational style (*P*=.045). This finding suggests that conversational style especially increases recall of information when presented audiovisually. This combination of modality and narration style improved recall of information among both younger and older adults.

**Conclusions:**

We conclude that combining audiovisual information with conversational style is the best way to present eHealth information to younger and older adults. Even though older adults did not proportionally recall more when audiovisual information was combined with conversational style than younger adults, this study reveals interesting implications for improving eHealth information that is effective for both younger and older adults.

## Introduction

### Background

Older adults have been one of the fastest growing groups using the Internet [1]. Recent figures show that the number of older adults using the Internet in Western countries, such as the United States and the Netherlands, has nearly doubled over the past few years [2,3]. More than half of these older adults used the Internet for accessing health information [3,4]. The Internet is seen as a relevant source of gathering health information among older adults [5] and has helped many older adults to fulfill a wide range of information and support needs [6,7]. However, simply having access to online health information (subsequently called eHealth information in this paper) does not necessarily mean that people find and understand such information. eHealth literacy, the ability to seek, find, understand, and act on health information from electronic sources to solve a health problem [8], is considered lower among older adults [9]. This may influence the extent to which health information is recalled. Recall of information is the ability to reproduce information and is crucial for optimal health outcomes, such as adequate disease management [10] and adherence to medical regimes [11,12].

Thus, effective message strategies need to be found to communicate eHealth information in such a way that older adults are able to recall information. However, previous research focusing on effective ways to present eHealth information to older adults in particular is lacking. The cognitive theory of multimedia learning (CTML) presents strategies that might enhance recall in general and could be applied to how older adults learn from eHealth materials. One strategy is to use audiovisual materials. The idea that people learn information more deeply from audiovisual materials compared to written materials is called the *modality effect* [13]. Another strategy described by the CTML is to use conversational narration style in messages. People are expected to learn information better when presented in conversational narration style rather than formal narration style, a phenomenon referred to as the *personalization effect* [14]. Surprisingly, to the best of our knowledge, no studies to date have examined the effects of combining modality (written vs audiovisual) and narration style (formal vs conversational) in eHealth messages on information recall. Combining such strategies might result in synergistic effects, which occur when the combined effect of these strategies exceeds the sum of their individual effects [15]. Furthermore, although previous research has shown support for modality and narration style strategies in younger adults [16,17], empirical evidence on the effects in older adults is particularly lacking.

The aim of our study is to identify effective ways of presenting Web-based information to enhance recall of eHealth information among older adults in particular. We test the (synergistic) effect of modality and narration style, and whether there are differences between younger and older adults. In assessing age differences, we chose participants younger than 65 years for the younger age group and 65 years and older as the older age group. These two age groups are generally considered suitable for separate analysis in aging research [18,19].

### Modality: Written Versus Audiovisual Information

The CTML attempts to explain how people learn and recall information in multimedia environments [20]. It is based on the assumption that people have separate information processing systems for visual and auditory materials [21] and that people are limited in the amount of material they can process in each of these systems at one time [22]. These assumptions are derived from the dual coding theory [21,22] and cognitive load theory [13]. The modality principle describes that one’s limited working memory capacity can be effectively expanded by addressing one or more sensory modalities, such as audiovisual information, instead of written information only [23]. A meta-analysis indeed showed an overall benefit in recall of information when information was presented in an audiovisual format compared to written format [16]. However, this meta-analysis did not include studies that were conducted in the field of eHealth information. We could nevertheless expect that audiovisual information would enhance recall of eHealth information as well. We therefore suggest H1a: Exposure to audiovisual information, compared to written information, has a positive effect on recall of eHealth information.

Whereas the modality effect among younger adults is repeatedly found in research [16], fewer studies have focused specifically on its effect among older adults [24]. The cognitive aging principle in multimedia learning [25] provides an integrative framework where the cognitive load theory is combined with general views of cognitive aging. This theory states that older adults’ limited working memory might be expanded by multimodal presentation of information. Older adults usually have a smaller “total cognitive capacity” than younger adults and might therefore benefit more from multimodal information than younger adults [26]. Previous research has demonstrated a benefit of multimedia messages for older adults by reducing cognitive load and decreasing learning time of information [24]. Even though this study did not present eHealth information to older adults, audiovisual information might be as effective for older adults when used in a Web-based context. We might therefore expect that audiovisual information positively influences older adults’ memory for eHealth information. Therefore, our second hypothesis H1b states: The effects of exposure to audiovisual information, compared to written information, on recall of eHealth information are greater in older adults than in younger adults.

### Narration Style: Formal Versus Conversational Style

Conversational style is often used in narrative communication, such as testimonials and personal stories or a description of an individual experience [27]. By presenting information from a first-person perspective using a more informal approach, it feels like the person in the message is talking to you [28]. However, health information has been traditionally presented in a formal style, presenting factual information, such as expressing professional opinions [29]. Unfortunately, a substantial number of people do not understand such formal health messages well enough to make informed decisions and act accordingly [30]. In an attempt to simplify health information, using a conversational style in narrative communication has shown to serve as an effective way of educating people about health [27,31]. Conversational style is more likely to be recalled because of conversational rules, such as commitment to try to understand a narrator’s story [32]. Moreover, conversational style has been found to increase recall of information among a variety of learner profiles, that is, different levels of education [33]. For this reason, we hypothesize H2a: Exposure to information in a conversational style, compared to information in a formal style, has a positive effect on recall of eHealth information.

In addition to the hypothesized effect of conversational style among both younger and older adults, it is expected that older adults may benefit in particular from having eHealth information presented in conversational style. Older adults tend to have better narrative recall and thus remember stories in more accurate detail than younger adults [34]. Using conversational style in eHealth information might therefore especially improve recall of information in older adults. Therefore, we expect H2b: The effects of exposure to information in a conversational style, compared to information in a formal style, on recall of eHealth information are greater in older adults than in younger adults.

### Synergistic Effects: Combining Modality and Narration Style

To our knowledge, no research to date has studied the synergistic effects of combining modality and narration style in enhancing recall of information. Nevertheless, various message strategies and styles are often combined in one health message, and it is likely that these message strategies have individual as well as synergistic effects [35]. For instance, one study found that tailoring Web-based information to individual characteristics was effective in realizing smoking cessation, however, only when information was presented in audiovisual format and not in written format [36]. This finding indicates that combining several message characteristics might optimize the effectiveness of a message. As we hypothesized that audiovisual information will outperform written information and conversational narration style will outperform formal narration style, we might expect H3a: Combining audiovisual information with conversational style, compared to other combinations of modality and narration style, has a positive effect on recall of eHealth information.

Moreover, as we hypothesized that especially older adults will benefit from audiovisual information and conversational narration style, we might also expect that combining these two might especially enhance older adults’ recall of information. Our final hypothesis H3b therefore states: The effects of combining audiovisual information with conversational style, compared to other combinations of modality and narration style, on recall of eHealth information are greater in older adults than in younger adults.

## Methods

### Design

For this experiment, a 2 (modality: written vs audiovisual information) by 2 (narration style: formal vs conversational style) between-subjects design was used. Age was assessed in the questionnaire and included as a factor: younger (<65 years) versus older (≥65 years) age. We used a Netherlands Cancer Institute (NKI) webpage where information about radio frequency ablation (RFA) treatment was given. RFA is a minimally invasive treatment, using radio frequency waves to destroy lung tumors.

Ethical approval for this study was granted by the Institutional Review Board of the Amsterdam School of Communication Research (reference number 2012-CW-33).

### Participants

Participants from the general population were selected from a large respondent pool by the ISO certified market research company PanelClix to participate in a Web-based survey. This Web-based setting allowed participants to engage in the experiment from their home computer, creating a natural and realistic setting to evaluate the stimulus materials. PanelClix made a random selection of their panel members to participate in the study stratified by age (<65 years vs ≥65 years). To be eligible to take part in the Web-based survey, participants had to be older than 18 years, able to read and write in Dutch, and should have no prior knowledge on RFA treatment, because this could influence the recall scores. An invitation for the study was available for PanelClix members on the PanelClix website. A total of 796 unique participants entered the survey, of which 788 unique participants started the survey (participation rate=99.0%), and 490 unique participants completed the Web-based survey (completion rate=62.2%). Duplicate entries were avoided by assigning pid-codes to participants, which were unique codes assigned to participants by PanelClix. Checking these pid-codes revealed 4 participants who completed the survey twice. In these cases, the second entry was excluded from the dataset (n=4). Participants who had prior knowledge on RFA treatment, that is, scoring higher than 4 on a 7-point Likert scale (n=23), were excluded as well. Since usability testing of the questionnaire showed that at least 10 minutes were needed to complete the survey, participants were excluded when they completed the survey in less than 10 minutes (n=15). Furthermore, participants were excluded when they viewed the experimental stimulus for less than 4 seconds (n=1), had not been exposed to the experimental stimulus due to technical issues (n=5), had used another source than the experimental stimulus to answer the recall questions (n=1), or reported to suffer from short-term memory loss (n=1). This resulted in a total of 440 participants included in the dataset.

### Procedure

When participants entered the Web-based survey, they were informed about the survey length, participants’ rights (eg, anonymity), and the contact details of the research institute and principle investigator. On the next page, participants were exposed to questions about gender, age, educational level, prior medical knowledge about lung cancer and RFA treatment, and Internet use. Based on age, participants were assigned to the younger or older age strata, and were randomly assigned to one of four experimental conditions. They were instructed to pay careful attention to the RFA information and could look at the information as long as they liked. Participants were not able to return to the written or audiovisual information (depending on condition) after continuing to the next page. The written information versions were presented on a webpage and the audiovisual information versions through a Web-based video. In the audiovisual conditions, participants were instructed to turn on their speakers. After exposure to the experimental stimulus, recall questions were shown. Upon completion, participants received credit points from PanelClix.

### Materials

Using the existing text (formal written condition, 245 words) of the NKI website, the conversational text was written (330 words). The formal audiovisual information was identical to the formal written information, and the conversational audiovisual information was identical to the conversational written information. The conversational versions were personalized in two ways: first, we changed the third-person perspective into a first-person perspective as if the narrator was talking to you [28] (eg, “A special needle guided by a CT scanner will be inserted into the cancerous lung tumor” vs “A special needle guided by a CT scanner was inserted into my cancerous lung tumor”). Second, we added sentences about personal experience to make the story more conversational as well (eg, “[…], depending on the location of the tumor” vs “I myself received sedation, but that depended on the location of the tumor”). In the formal audiovisual conditions, a doctor was videotaped behind a desk and in the conversational audiovisual conditions, a patient was filmed sitting on a couch. The same actors starred as the doctor and patient to ensure that narration style effects could be attributed to changes in narration style rather than changes in narrator. In addition, both the formal and conversational audiovisual information was filmed twice to create a formal and conversational video starring a younger narrator as well as a formal and conversational video starring an older narrator. This resulted in four videos in which information was presented by (1) a younger doctor (2:01 min; see Multimedia Appendix 1), (2) an older doctor (2:05 min; see Multimedia Appendix 2), (3) a younger patient (2:53 min; see Multimedia Appendix 3), or (4) an older patient (2:34 min; see Multimedia Appendix 4). This was done to control for potential identification effects with a specific narrator because of age similarity. However, there were no differences in identifying with the younger and older actor (*F*_1,245_=0.06, *P*=.86, η_p_^2^=0.00), regardless of participants’ own age (*F*_1,245_=0.13, *P*=.72, η_p_^2^=0.00). Therefore, this additional design factor was not considered while analyzing the data. Thus, four experimental conditions were analyzed: (1) written information in formal style, (2) written information in conversational style, (3) audiovisual information in formal style, and (4) audiovisual information in conversational style. Compared to previous research on the effects of modality and narration style, our materials were similar in terms of length [16,17].

### Measures

#### Background Characteristics

Background measures included participants’ age, gender, level of education, Internet use, and prior medical knowledge. Education was divided into low level of education (primary education, lower vocational education, preparatory secondary vocational education, and intermediate secondary vocational education), middle level of education (senior secondary vocational education and university preparatory vocational education), and high level of education (higher vocational education and university). Internet use was assessed through average number of hours spent per week on the Internet. Prior medical knowledge about lung cancer in general and RFA knowledge specifically was measured by two items asking how much knowledge participants perceived to have about lung cancer and RFA treatment respectively using a 7-point scale (1=no knowledge at all, 7=very much knowledge).

#### Recall of eHealth Information

Information recall was measured using the Netherlands Patient Information Recall Questionnaire [37]. Questions were created from the RFA information and were pretested among 12 students. This resulted in 11 open-ended recall questions, such as “Could you please name the most common complication during an RFA treatment?” All questions were accompanied by a textbox for participants to provide their answer. Recall scores could range from 0 (not recalled), to 1 (recalled partially), to 2 (recalled correctly). A codebook was used for allocating scores to each of the recall questions. Two independent coders used this codebook and double coded 14.1 % (62/440) of the recall scores. Interrater reliability appeared to be good (mean kappa=.84, range 0.65-1.00). The 11 items were computed into a total recall score, ranging from 0-22. Additionally, percentages of the recall scores were calculated by dividing the participant’s total score by 22.

### Statistical Analysis

For testing successful randomization, analyses of variance and chi-square tests were conducted to check for unequal distribution of background variables across the 2 (modality) × 2 (narration style) × 2 (age groups) experimental design. Educational level and medical knowledge about lung cancer were found to be unequally distributed (respectively χ^2^_14_=24.72, *P*=.04 and *F*_7,432_=2.06, *P*=.047, η_p_^2^=0.03). Moreover, previous studies have identified educational level and prior topic knowledge as important predictors of information recall [38]. We therefore included educational level and medical knowledge about lung cancer as covariates in all analyses. For testing the main effects of modality and narration style, an ANCOVA (analysis of covariance) was conducted with recall of information as dependent variable, modality, narration style, and age groups as factors, and education and medical knowledge as covariates. The synergistic effects between modality and narration style were tested in an ANCOVA using one variable for modality and narration to measure the simple contrast effects between conversational audiovisual information and the three other combinations of modality and narration style. To test whether the effect of modality and narration style was greater among older adults than younger adults, the data file was split on age group and the above described analyses were repeated.

## Results

### Sample Characteristics

Of the participants who filled out the Web-based questionnaire (53.9%, 237/440 male and 46.1%, 203/440 female), 53.6% were younger (236/440; <65 years; mean 41.78, SD 12.69) and 46.4% were older (204/440; ≥65 years; mean 69.44, SD 4.13). Besides age (*F*_1,438_=887.88, *P*<.001, η_p_^2^=0.67), the two age groups significantly differed in educational level (χ^2^_2_=18.61, *P*<.001; see Table 1 for sample characteristics).

**Table 1:** Sample characteristics (N=440).^a^

		Younger adults (<65 years) (n=236)	Older adults (≥65 years) (n=204)
**Gender, n (%)**			
	Male	123 (52.1)	114 (55.9)
	Female	113 (47.9)	90 (44.1)
Age in years, mean (SD) range		41.78 (12.69) 18-64	69.44 (4.13)^b^65-85
**Education level, n (%)**			
	Low	50 (21.2)	81 (40.1)^b^
	Middle	104 (44.1)	69 (34.2)^c^
	High	82 (34.7)	52 (25.7)
Internet use (hours per week), mean (SD) range		18.21 (11.72) 1-60	17.83 (11.59) 1-60
Medical knowledge about lung cancer^d^, mean (SD) range		2.88 (1.41) 1-6	2.66 (1.37) 1-7
Medical knowledge about RFA^d^, mean (SD) range		1.71 (1.06) 1-4	1.54 (0.94) 1-4

^a^Not all figures add up to 100% due to missing data. Conditions stratified by age only significantly differed on education.

^b^Differs significantly from younger adults (*P*<.001).

^c^Differs significantly from younger adults (*P*=.04).

^d^A higher score indicates more knowledge, ranging from 1 to 7. RFA: Radio Frequency Ablation.

### Effects of Modality on Recall of eHealth Information

The first hypothesis predicted a positive effect of audiovisual information (vs written information) on recall of eHealth information (H1a), which was greater for older adults in particular (H1b). Modality significantly influenced recall of health information (*F*_1,413_=4.48, *P*=.04, η_p_^2^=0.01). As hypothesized, the audiovisual conditions (mean 7.60, SE 0.31, 95% CI 7.00-8.21) resulted in significantly higher recall scores than the written conditions (mean 6.55, SE 0.39, 95% CI 5.79-7.32). However, older adults did not recall more from audiovisual information compared to written information (*F*_1,189_=1.59, *P*=.21, η_p_^2^=0.01) than younger adults (*F*_1,223_=3.09, *P*=.08, η_p_^2^=0.01). These results, thus, partially confirm our first hypothesis. Recall scores across modality and age groups appear in Table 2.

**Table 2:** Main effects of modality on recall of eHealth information in younger and older adults.^a^

Group		n^b^	Recall of health information		
			% recall	Mean (SE)	95% CI
**All participants**					
	Written information	165	29.8	6.55 (0.39)	5.79-7.32
	Audiovisual information	273	34.5	7.60^c^(0.31)	7.00-8.21
**Younger participants**					
	Written information	85	28.2	6.20 (0.55)	5.12-7.27
	Audiovisual information	151	34.1	7.50 (0.42)	6.68-8.32
**Older participants**					
	Written information	80	31.4	6.91 (0.55)	5.83-7.99
	Audiovisual information	122	35.0	7.70 (0.45)	6.82-8.59

^a^Adjusted for education level and medical knowledge about lung cancer. Recall of information ranges from 0-22. Percentage of correct recall is based on mean scores divided by 22. The higher the score, the more information was recalled correctly.

^b^The category sizes differ because of 2 fewer cases due to missing covariate values.

^c^Differs significantly compared to written information (*P*=.04).

### Effects of Narration Style on Recall of eHealth Information

Our second hypothesis predicted a positive effect of conversational narration style (vs formal narration style) on recall of eHealth information (H2a), which was expected to be greater for older adults in particular (H2b). However, no main effect of narration style on recall of information was found (*F*_1,413_=1.89, *P*=.17, η_p_^2^=0.01). The means in Table 3 show that using conversational style does not significantly increase recall of information (mean 7.42, SE 0.35, 95% CI 6.74-8.10) compared to formal style (mean 6.74, SE 0.35, 95% CI 6.04-7.43). Furthermore, older adults did not recall more information when conversational style (vs formal style) was used (*F*_1,189_=1.78, *P*=.18, η_p_^2^=0.01) compared to younger adults (*F*_1,223_=0.51, *P*=.48, η_p_^2^=0.00). This was not expected and, therefore, our second hypothesis was rejected. Recall scores across narration style and age groups are shown in Table 3.

**Table 3:** Main effects of narration style on recall of eHealth information in younger and older adults.^a^

Group		n^b^	Recall of health information		
			% recall	Mean (SE)	95% CI
**All participants**					
	Formal style	214	30.6	6.74 (0.35)	6.04-7.43
	Conversational style	224	33.7	7.42 (0.35)	6.74-8.10
**Younger participants**					
	Formal style	119	30.0	6.61 (0.48)	5.67-7.55
	Conversational style	117	32.2	7.09 (0.50)	6.11-8.06
**Older participants**					
	Formal style	95	31.2	6.86 (0.52)	5.84-7.88
	Conversational style	107	35.2	7.75 (0.48)	6.80-8.70

^a^Adjusted for education level and medical knowledge about lung cancer. Recall of information ranges from 0-22. Percentage of correct recall is based on mean scores divided by 22. The higher the score, the more information was recalled correctly.

^b^The category sizes differ because of 2 fewer cases due to missing covariate values.

### Synergistic Effects

**Figure 1:**
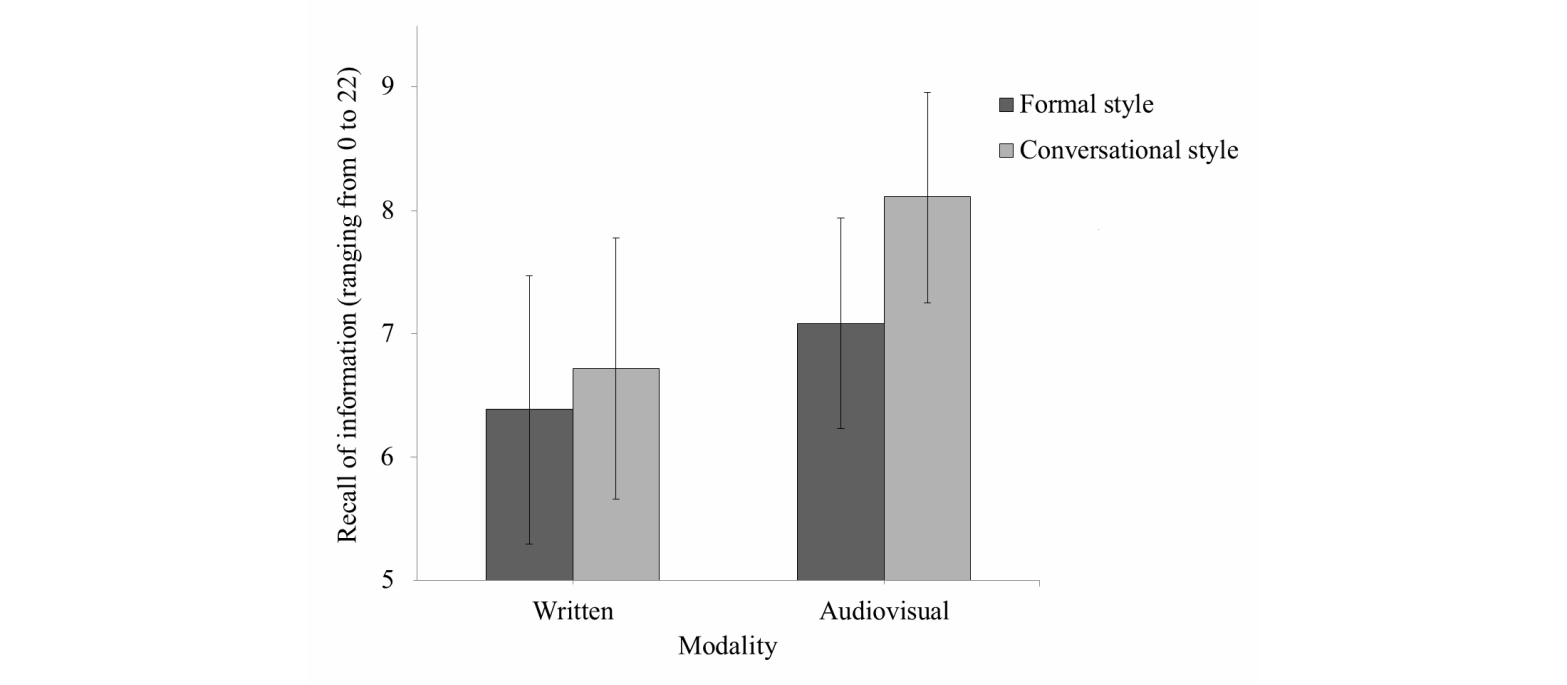
Combining audiovisual information with conversational narration style results in highest recall of eHealth information among younger and older adults. The bars and error bars represent the mean recall scores and 95% confidence intervals respectively.

Our third hypothesis concerned the synergistic effect between modality and narration style (H3a), which was also expected to be greater for older adults (H3b). We expected that combining audiovisual information with conversational style would outperform other combinations of modality and narration style. In support of our hypothesis, we found that combining audiovisual information with conversational style resulted in the most favorable recall scores compared to combining written information with formal style (contrast estimate=-1.73, SE 0.70, *P*=.01, 95% CI -3.11 to -0.35]), as well as compared to combining written information with conversational style (contrast estimate=-1.40, SE 0.70, *P*=.045, 95% CI -2.76 to -0.03). This finding suggests that conversational style especially increases recall of information when presented as audiovisual information, but not when presented as written information (see Figure 1).

The expected synergistic combination of audiovisual information and conversational style (eg, vs combining written information with formal style) did not particularly improve recall of information among older adults (contrast estimate=-1.73, SE 0.95, *P*=.07, 95% CI -3.60 to 0.15) compared to younger adults (contrast estimate=-1.80, SE 1.03, *P*=.08, 95% CI -3.84 to 0.24). Our data therefore partially confirm our third hypothesis. The synergistic effects between modality and narration style on recall of information (for all participants and stratified by age group) are provided in Table 4.

**Table 4:** Synergistic effects of combining modality and narration style on recall of eHealth information in younger and older adults.^a^

Group			n^b^	Recall of health information		
				% recall	Mean (SE)	95% CI
**All participants**						
	**Written**					
		Formal	82	29.1	6.39 (0.56)	5.29-7.48
		Conversational	83	30.6	6.72 (0.54)	5.65-7.79
	**Audiovisual**					
		Formal	132	32.2	7.09 (0.44)	6.23-7.94
		Conversational	141	36.9	8.12^c,d^(0.43)	7.26-8.97
**Younger participants**						
	**Written**					
		Formal	44	28.0	6.16 (0.77)	4.66-7.67
		Conversational	41	28.3	6.23 (0.78)	4.69-7.77
	**Audiovisual**					
		Formal	75	32.1	7.06 (0.57)	5.94-8.18
		Conversational	76	36.1	7.94 (0.61)	6.73-9.15
**Older participants**						
	**Written**					
		Formal	38	30.1	6.61 (0.81)	5.03-8.19
		Conversational	42	32.8	7.21 (0.75)	5.73-8.68
	**Audiovisual**					
		Formal	57	32.4	7.12 (0.66)	5.83-8.41
		Conversational	65	37.7	8.29 (0.61)	7.09-9.49

^a^Adjusted for education level and medical knowledge about lung cancer. Recall of information ranges from 0-22. Percentage of correct recall is based on mean scores divided by 22. The higher the score, the more information was recalled correctly.

^b^The category sizes differ because of 2 fewer cases due to missing covariate values.

^c^Differs significantly compared to formal written information (*P*=.01).

^d^Differs significantly compared to conversational written information (*P*=.045).

## Discussion

### Principal Findings

In this study, we aimed to identify effective ways of presenting eHealth information to enhance recall of information among older adults in particular. We examined the (synergistic) effect of modality and narration style on recall of health information, and whether there are differences between younger and older adults. Our results support the modality effect as proposed by the CTML [13]. Younger and older individuals who were exposed to audiovisual information recalled more health information than those who were exposed to written information. Our findings do not show support for the personalization effect, another principle proposed by the CTML [14]. Health information was not better recalled when presented in conversational style than in formal style. However, combining audiovisual information with conversational style led to the highest recall scores, outperforming combining written information with formal style and written information with conversational style. This underscores that conversational style is especially effective in improving recall of health information when presented audiovisually, rather than when presented as written information.

Even though we found that older adults benefited from audiovisual information with respect to better recall of information, they did not proportionally benefit more than younger adults, as proposed in the cognitive aging principle in multimedia learning [25]. This might be explained by the fact that audiovisual information is not self-paced, whereas written information is. Previous research has indicated that self-pacing of information plays an important role in older adults’ recall of information [39]. When older adults have the option to self-pace information, they are able to take the time they need to process information, which may result in information recall that is comparable to that of younger adults (personal communication by Bol, Van Weert, Loos, Romano Bergstrom, Bolle & Smets, 2014). Likewise, another study showed that older adults need more time than younger adults to recall equivalent amounts of information [40]. Hence, older adults might benefit most from self-paced information. However, audiovisual information is traditionally not self-paced. A recent experimental study, in which self-paced written information was compared with self-paced spoken information, revealed that self-paced spoken information outperformed self-paced written information in older adults with limited health literacy [41]. When exposed to self-paced spoken animations (ie, spoken information with simulated motion pictures), older adults with low health literacy recalled the same amount of information as their high health-literate counterparts. Older people with health disparities might therefore especially benefit more from audiovisual information with respect to better recall. As health literacy was not measured, and individuals with limited health literacy might have been underrepresented in the current sample, this seems worth investigating in future research.

This study provides evidence for effective Web-based communication strategies by revealing the promising effects of combining message factors, such as audiovisual information and conversational narration style, to enhance information recall in both younger and older adults. In addition to this practical contribution, the results of this study also add to the current synergy literature. Our results suggest that synergistic effects do not occur only at the level of combining multiple media as suggested by Naik and Raman [15], but also at the level of combining multiple message characteristics, in this case, modality and narration style. Nonetheless, we need to use caution in generalizing these results as there are more message characteristics to explore and effective combinations to discover (for an overview of other message characteristics, see [28]).

### Limitations

This study has several limitations. First, the manipulation we used for narration style could be considered as a study limitation. In Web-based written material, conversational style can be clearly manipulated by changing passages in the text [28]. However, when working with audiovisual information, we dealt with visual changes as well, that is, changing the type of narrator (doctor vs patient). Having a doctor versus a patient explain information changes source attributes, which might have changed the perceived source expertise and the perceived level of authority [42]. This change might have confounded the association between conversational style and recall of information in the audiovisual conditions, providing an alternative explanation for the synergistic effect found when combining audiovisual information with conversational style. Furthermore, it should be noted that simply putting written information into spoken format will not fully ensure equivalence. Features that determine the listenability of spoken messages are not the same as the features that determine the readability of written messages [43].

Second, although combining audiovisual information with conversational narration style led to the highest recall scores, it is important to bear in mind that the overall recall scores in this study were low. It has been estimated that 40%-80% of medical information is immediately forgotten [44], which has also been found with regard to recall of Web-based medical information [45,46]. One explanation for the low recall scores in this study could be that the RFA information presented was rather complex. However, it was the original RFA information presented on the website of a specialized cancer hospital, and we know that the majority of eHealth information is complex [47]. As message complexity is associated with poor recall of information [46], it is crucial to consider message complexity when designing eHealth materials to improve recall of eHealth information

Third, we did not include the target sample for testing our stimulus materials, that is, cancer patients. Patients might have been more involved and motivated because of their personal experience with cancer and seeking Web-based cancer information. As involvement has often been associated with deeper processing of information [48], including cancer patients might have resulted in higher recall scores than found in the current study. Furthermore, the younger and older adults in our healthy study sample did not differ in their Internet use. Even though older adults are increasingly using the Internet [1], they are still using the Internet considerably less than their younger counterparts [3]. This bias might explain the lack of age differences found in this study. Nevertheless, including healthy individuals still resulted in support for effectively combining audiovisual information with conversational style (vs written information), and these effects might even increase when tested among cancer patients and individuals less experienced with the Internet.

### Implications and Future Research Directions

This study adds to the literature by applying modality and narration style strategies to the field of eHealth information. Dealing with eHealth information, especially when it concerns life-threatening diseases such as cancer, often involves feelings of stress and anxiety [49]. In an attempt to reduce such feelings, individuals use different coping strategies [50]. For instance, “monitors” intentionally seek information to reduce stress and “blunters” avoid information to diminish stress [50]. It is recommended to focus on effective communication strategies in the field of eHealth information. Future research could benefit from including cancer patients in such research to examine how coping style interferes with learning material that is emotionally demanding and stressful.

Although the recall scores of both younger and older adults were low, future research should also be aimed at getting more insight into the effectiveness of eHealth information by focusing on what message strategies work best for older audiences in particular. Older adults are often vulnerable to poor Web-based communication, due to their limited eHealth literacy and inexperience with Internet technologies [9]. In the current climate of presenting crucial health information through the Internet, it is important to consider aging populations when designing eHealth information. By better understanding how older adults process Web-based information, more successful eHealth information can be developed based on successful combinations of message characteristics, rather than providing a best guess combination. Previous research has highlighted the importance of recognizing the diversity of message preferences among older adults [51], which should be considered when developing eHealth materials for this older age group. Future research should focus on such (age-related) factors that might impact how eHealth information is remembered to optimize eHealth tools.

## Multimedia Appendix 1

The formal audiovisual message in which a younger doctor presents information.

## Multimedia Appendix 2

The formal audiovisual message in which an older doctor presents information.

## Multimedia Appendix 3

The conversational audiovisual message in which a younger patient presents information.

## Multimedia Appendix 4

The conversational audiovisual message in which an older patient presents information.

## Abbreviations

ANCOVA: analysis of covariance
CTML: cognitive theory of multimedia learning
RFA: radio frequency ablation
